# Environmental pediatric physiotherapy and risky play: making the case for a perfect match

**DOI:** 10.3389/fpubh.2024.1498794

**Published:** 2025-01-22

**Authors:** Andrea Sturm, Ellen Beate Hansen Sandseter, Barbara Scheiber

**Affiliations:** ^1^Department of Physiotherapy, Health University of Applied Sciences Tyrol, Innsbruck, Austria; ^2^Queen Maud University College of Early Childhood Education, Trondheim, Norway

**Keywords:** physiotherapy, environmental, child health, public health, risky play, physical therapy, pediatric

## Abstract

Environmental physiotherapy is epistemologically anchored in the critical recognition that physiotherapeutic practice is fundamentally embedded within a planetary ecological framework, demanding a holistic, systemically integrated approach to professional practice. This perspective article highlights and underscores the value of risky play for child health and the commonalities with environmental pediatric physiotherapy. The article starts with a discussion of current challenges in child health around the globe, often resulting from a lack of physical activity of children, and claims finding new, promising and sustainable ways that are able to attract children and their parents to playfully increase the time that children are physically active. Followed by an overview of physiotherapists’ roles and responsibilities in child public health, the authors point to the need to move beyond an isolated profession-centric approach when tackling the existing, concerning issues in child health worldwide. Foundational information about risky play underpinned with scientific results and its acknowledgment by other health professions is then presented. By including a perspective of what children want, the authors identify a gap between the world’s children’s actual needs and current societal offers. The benefits of risky play for child health are presented in detail, along with a discussion of various considerations pertaining to child safety. Concluding, this perspective article demonstrates how physiotherapists can contribute to better child health by including risky play in physiotherapy theory and practice.

## 1 Introduction

About 80% of the world’s youth does not meet the recommendations of the World Health Organization (WHO) to be physically active for at least 60 min per day (1, 2), contributing to a global pandemic of physical inactivity, which is considered to be a major cause of global mortality (1–4). According to the WHO, in 2022, 37 million children under the age of 5 and 390 million children between 5 and 17 years old were overweight or obese, with increasing numbers in high-, middle-as well as in low-income countries (4, 5). One of the multifactorial reasons for this unfavorable development is insufficient levels of physical activity in children and adolescents (6), an accelerant for an early onset of non-communicable diseases (NCDs) such as diabetes type 2, cardiovascular diseases, neurological and mental health disorders, cancers, chronic respiratory diseases, or eating disorders (5, 7). Children with disabilities are even more predisposed to developing secondary health conditions (8).

Physical activity is clearly related to better health outcomes and recognized to be one of the most efficient ways to enhance a person’s overall health across their lifespan (9–12). A decrease of 15% in physical inactivity worldwide is targeted by the WHO Global Action Plan on NCDs, by 2030. However, the implementation of physical activity on national levels is still insufficient (13). Although there is enough research proving the health benefits of physical activity, conducted over decades, the number of NCDs still increases, and rates of physical activity are changing slowly. Do we miss something important? Of course, better infrastructures, more resources for physical activity, as well as effective coalitions of multiple sectors are important for achieving more meaningful results (1, 14). But considering the huge amount of children and adolescents identified as being physically inactive around the globe (15), new, effective, and low-threshold strategies of health promotion are required to address these concerning developments in child health. It has been identified as a worldwide priority to develop approaches to target these concerning issues that are accessible, acceptable, cost-effective, culturally adaptable, feasible and to ensure an optimal compliance—of course fun (3, 16–19).

There is a tendency for physical activity and sedentary behavior habits learned during childhood to persist into adulthood (20). This highlights the importance to find joyful and meaningful ways for children, at an early age, to bodily movement and being physically active. Children with disabilities and of course also those without disabilities experience physiotherapy more positively when it is engaging, fun and pleasurable (21). Promoting physical activity in children should enable a broader health experience, including a lively child-environment interaction (22), which contributes to satisfaction, enjoyment, perceptions of social inclusion, and self-efficacy (23). Playful physical activities are promising to reach a large group of children, allowing them to explore, develop and enhance their physical abilities, motor and social skills, sense of belonging, self-efficacy and self-awareness, as well as to playfully integrate and overcome their fears. Not only is a child’s right to play enshrined by Article 31 of the United Nations Convention on the Rights of the Child (24), play is necessary for child development. It is considered to be therapeutic, an instinctive mode of self-directed learning and efficient means of teaching and guidance, and a contribution to and enactment of health and wellbeing (25). Interestingly, the UN’s general comment on the fulfillment of Article 31 has identified an increased safety focus and a lack of access to nature as threats to children’s free play (26).

Physiotherapists are well-situated to mitigate these threats impacting child health. They are autonomous experts (27), tailoring individualized activity programs aligning with each child’s unique motor developmental profile and functional capabilities (28). Physiotherapists engage with children and their families during developmental stages when health-related behaviors are amenable to intervention and modification and are expected to actively advocate for innovative strategies that foster health prevention and physical well-being among children throughout their lifespan (28–30). They work interdisciplinary in clinics, schools, communities, early childhood centers and hospitals, provide child and family-focused education, and identify and connect families with resources for health promotion and motor skill acquisition (28). Physiotherapists play an active role in bringing the United Nation’s Agenda 2023 Sustainable Development Goals (SDGs) into life (31–34), create effective interdisciplinary coalitions to implement the WHO Global Action Plan on NCDs (1) and transcend biomedical perspectives on child health and disability into holistic perspectives by applying the WHO’s International Classification of Functioning, Disability and Health for Children and Youth (ICF-CY) (22, 35, 36). In this perspective article, we aim to highlight both, the value of risky play for child health and the worth of including this pedagogical stance into the provision of physiotherapy services. Contributing to this special issue on reframing the role of the physiotherapy profession in the Anthropocene, we will put weight on outdoor risky play during early childhood. We outline the well documented benefits of risky play for child health and overall development, and claim more and broader recognition of these benefits by the physiotherapy profession. Additionally, we advocate for future research investigating the interplay of physiotherapy, risky play and child health and the inclusion of knowledge about risky play in physiotherapy curricula to support contemporary pediatric physiotherapy.

## 2 Risky play

Risky play is defined as thrilling and exciting forms of play that involve a potential risk of injury (37), with ambiguity and uncertainty being understood as important characteristics (38). Risky play can happen indoors but is most common in outdoor settings where children are allowed to play freely (39, 40). Eight categories of risky play (see Table 1) have been identified through observations and interviews with children and adults (41, 42). Most of the research on children’s risky play has been situated within the context of early childhood education, focusing on 1 to 6-year-olds. Children seek thrills and risks in their play and on a level that suits their individual competence and courage (43–45). Research shows that risky play is a common type of play both among girls and boys and across ages in the early years (39, 41, 42). Taking risks in play is also strongly connected to children’s risk management skills. Research identifies how children are aware of the risk they are taking and how they mitigate or increase the risk according to their own skills and how they experience the situation (46, 47), and in addition, how risky play contributes to increased risk assessment skills (48).

**Table 1:** Eight categories of risky play, including related risks and sub-categories.

Category	Risk	Sub-categories
A: Great heights	Danger of injury from falling	Climbing Jumping from still or flexible surfaces Balancing on high objects Hanging/swinging at great heights
B: High speed	Uncontrolled speed and pace that can lead to collision with something (or someone)	Swinging at high speed Sliding and sledging at high speed Running uncontrollably at high speed Bicycling at high speed Skating and skiing at high speed
C: Dangerous tools	Can lead to injuries and wounds	Cutting tools: knifes, saws, axes Strangling tools: ropes, etc.
D: Dangerous elements	Where children can fall into or from something	Cliffs Deep water or icy water Fire pits
E: Rough-and-tumble	Where the children can harm each other	Wrestling Fencing with sticks, etc. Play fighting
F: Disappear/get lost	Where the children can disappear from the supervision of adults, get lost alone	Go exploring alone Playing alone in unfamiliar environments
G: Play with impact	Where children are crashing—either themselves or an object—into something	Throw themselves onto a mattress Crash their tricycle into a fence
H: Vicarious risk	Where the risk is only observed by the children	Watching other children’s risky play for a length of time

Over the years, eight categories of risky play have been identified. By investigating various kinds of risky play through interviews and observations of preschoolers and staff in Norway, Sandseter (41) identified six categories of risky play (A–F). Kleppe et al. (42) identified two more categories of risky play of toddlers aged 1–3 years (G, H).

In children with disabilities, the understanding of risk-taking and risky play is wider and more inclusive and does not contradict the original intention of the concept of risky play as being unstructured, child-driven activities (49). In a recent position statement, the Canadian Pediatric Society underscored the value of risky play for children’s healthy physical, mental, and social development (50) due to its potential to prevent injuries (51, 52) by children learning how to manage risks and tackling health problems such as poor development of motor functions, obesity, anxiety, and behavioral issues (9, 50, 53–56). Research showed that physical activity is 2.2 to 3.3 times higher when children are playing outdoors than indoors, independent of different age groups, sexes, and contexts (16). Children spend significantly more time being active in adventurous environments than where structures are pre-fabricated (55). Furthermore, natural environments provide the ideal conditions for children, regardless of varying developmental levels, to engage in challenging and exciting forms of playful physical activity (38, 57–59). Unfortunately, children’s opportunities to play freely outdoors are decreasing. The reasons for this can be grouped into four categories: time (nature-starved curriculum, time-poor parents, lack of time for free play), fear (stranger danger, dangerous streets, risk-averse culture); technology (rise of screen time); and space (vanishing green space) (60). Recently, the negative consequences of the rise of social media have been discussed in depth, pointing to risky play as an effective way to ensure ideal childhood development for current and future generations (61). Within the physiotherapy profession, the Australian Physiotherapy Association (APA) advocated for risky play as a response to school lockdowns during the COVID-19 pandemic (62), and an online course for rehabilitation professionals has been released to highlight the benefits of risky play for child health (63).

### 2.1 What children want

Children prefer playing with natural materials and in natural environments over screen time (25, 38). If children have the opportunity to experience nature, they develop a sense of care and a desire to conserve it (64). The experience of fun has been identified as a central experience for children, found within all physical and social environments. Children seek intense play experiences, want to make their own choices about what to play, and want to belong to their playgrounds (57). Obviously, what children around the globe mostly want and long for, differs more and more from what the societies they live in offer them: car-oriented, urbanized and increasingly highly digitalized, rather risk-averse societies as well overprotective environments, resulting in social encounters which rather isolate from each other than bringing communities actively together (61, 65), further contributing to nature-deficit disorders (66). Current generations of children spend less time playing outside and do so less frequently compared to their parents’ generation (64, 67, 68). There is a shift in children’s physical activity away from unsupervised and unstructured outdoor play toward structured and supervised activities that are primarily performed indoors (16, 18).

### 2.2 Why is risky play so important for children?

Engaging children in risky play is one of the best ways of injury prevention because their experiences lead to the ability to manage risks (52, 53). Children acquire coping mechanisms to handle risky situations by engaging in playful methods to evaluate and conquer risks, adapting to failure and adverse outcomes of their choices, leading to resilience and self-sufficiency (69). Risky play is naturally not only observed in children of all cultures but is found in many mammal species, representing an adaptive function (53, 54) by supporting the offspring in developing cognitive, emotional, motor, functional and social abilities. Playing freely plays an important role in the neurophysiological maturation of the prefrontal cortex, which influences self-regulation of behavior and impulsiveness and encourages self-reflection (56, 70–72). Risky play is considered to have an anti-phobic effect on a child’s physiological development, where a child will first show normal adaptive fears, which protect it against various risk factors. Risky play is a stepwise fear-reducing behavior. Hindering children from taking part in age-adequate risky play may result in increased neuroticism or psychopathology in society, as the fears may continue despite being no longer relevant due to a child’s physical and psychological maturation, possibly even turning into anxiety disorders (56).

The rising anxiety about children’s safety has been described as a common feature of modern societies. Besides overprotective parenting, also the conception of childhood has changed (65). The understanding of a child as resilient and capable shifted to the picture of a vulnerable child who needs continuous safeguarding, although this may not be true for all children and societies (73). Physiotherapists invest a great deal of time and effort by educating patients and the public to combat such misleading beliefs that can influence an individual’s lifestyle choices and quality of life (74, 75). Indeed, research shows that toddlers have the ability to assess and manage potential risks when they play outside in natural environments. Even 4 to 5-years old children were reported to be able to balance their risk-taking decisions between their evaluation of positive and negative outcomes of the play (76). Toddlers’ engagement in risky play promotes a sense of belonging through shared engagement in risky play experiences and by developing a personal connection with the place. Furthermore, social belonging to the group becomes evident when children demonstrate care and support towards each other when children’s peers stumble and fall whilst navigating through challenging environments (77).

Preschoolers spend up to twice as much time being sedentary indoors than when they are outdoors. The longer the time that children are outside, the more active they are (16). Natural landscapes play a significant role in the motor development of children, as gross motor functions such as running, jumping, throwing, climbing or crawling are predominant when children actively play in nature (78). Outdoor activity in children is related to lower diastolic blood pressure and to greater aerobic fitness (16). Additionally, nature-based activities in early childhood increase the diversity of children’s gut microbiome (79, 80), potentially reducing the risk of several immune-mediated diseases such as allergies, asthma, and type 1 diabetes (80), increase connectedness to nature, and may decrease perceived stress and anger frequency (79). Children with greater independent mobility meet more often to play with their peers, schoolmates and neighbors than children with less independent mobility (risky play category disappear/get lost, see Table 1). Physical activity outdoors also includes active transportation to commuting to school or other places (81), either by walking or riding a bike. The percentage of children using active transportation to get to and from places differs greatly between and among countries. The more developed the countries are, the lower they score in active transportation of children. In developing countries, active transportation may be the result of a lack of access to public transport and motor vehicles, but highly developed countries scoring high in active transport of children provide both infrastructure and policy to support active transportation (15). Rough and tumble play (see Table 1) is associated with higher interpersonal cognitive problem-solving in boys, but not with increased aggression (55). And, while we are on the subject of misleading convictions: the assumption that leaving children indoors is safer than outdoors may be wrong considering the potential harms of the internet including online-violence, cyber-bullying, pornography or image based sexual abuse (82), increased sedentary time and unnecessary incidental eating (18). These facts—both the encouraging and concerning ones—call for action.

What can physiotherapists do that current and future generations of children—the world’s later adults—are able to live how they wish, to gain the experiences that they need, and can build nourishing relationships with their bodies, their peers, their neighborhood, and their natural environment?

## 3 How physiotherapists can contribute

Restricted opportunities for outdoor and risky play were discussed as having a potentially negative impact on children’s physical activity behavior (55). Therefore, increasing the amount of time children spend outdoors seems to be a promising strategy to increase their physical activity levels and to promote healthy, active lifestyles (16) that persist into adolescence and adulthood. Physiotherapists are equipped with essential knowledge about both optimal childhood development and how various pediatric conditions can affect a child’s growth across multiple domains—including motor function, sensory processing, cardiorespiratory function, cognitive skills, and socio-emotional wellbeing (30). They are trained to autonomously choose and use a range of assessment approaches and therapeutic interventions, tailored to the child’s age and stage of development, and provide guidance to caregivers on how to create safe(-as-necessary) environments for their children (19, 29, 83). Therefore, physiotherapists are predestined to spread the word about the benefits of risky play for optimal child development and health prevention, and tackling existing health issues (84) by including elements of risky play (see Table 1) into pediatric physiotherapy interventions, where appropriate.

Around the globe, physiotherapists are well-situated in reaching out to large groups of children and their caregivers, serving diverse communities including minority cultural groups, immigrants/refugees, persons living with disabilities, and gender-diverse communities. They can translate elements of risky play into various environments and cultures (69, 85), contributing to justice (86, 87) and pleasurable, positive experiences and achievements, which are valued by children and their caregivers (21). The International Organization of Physical Therapists in Pediatrics (IOPTP) firmly recommends that pediatric physiotherapy education programs include content focused on health promotion, injury prevention and physical fitness (30)—all realms positively influenced by risky play. In some countries, due to legal regulations or a culture of litigation, supervising adults are concerned about potential lawsuits, when stretching their own limits pertaining to risk-taking in child play (69, 88). Working collaboratively with caregivers, educators, and other health professionals, as well as policymakers, legislators, and other stakeholders in child health and safety, physiotherapists can inform about the benefits of risk-taking in physical activities, to change the negative perception of risk in children’s play (see Table 2) (83, 84, 89). For example, the promotion of better public understanding of risky play’s importance for children’s health and development can be done through public talks, conferences, and interdisciplinary discussions, helping society to better understand why risky play matters and by advocating for environments and conditions that support it.

**Table 2:** Risks vs. hazards.

Risk in the context of risky play is understood as a situation whereby a child can recognize and evaluate a challenge and decide on a course of action how to respond to the challenge. The negative interpretation of risk led to a view of risk and hazard as being synonymous (53). A risk is not necessarily just negative. It can also be defined as situations in which we have to make choices among alternative courses of action without knowing the outcome (53). The presence of adults supervising children decreases injury rates. By enabling children to make choices that entail a risk of injury, while a caring adult helps to explore potential outcomes, their comprehension of the functioning of the world expands, fostering their development into independent and capable decision makers (55, 94).	A hazard is understood as a danger in the environment that could seriously injure or endanger a child and is beyond the child’s capacity to recognize (53). Considering the developmental and physical literacy skills of children engaging in risky play, risks that may pose hazards are eliminated from the play environment, taking into account the child’s age (53). Sometimes a hazard cannot be removed. In some regions of the world, children grow up in war-zones, where land-mines could be a severe threat to their lives, or they live in regions where encounters with wild or venomous animals are common. Extreme weather events such as heat or cold, long-term health risks of sun-exposure, or air pollution may impact a child’s ability to play outside without restrictions. Depending on the society one lives in, both high levels of traffic, or even violence, can contribute to an unsafe environment for a child to freely play outside.

Physiotherapists can initiate multidisciplinary research collaborations to investigate the health benefits of risky play in various community settings, environments and cultures, raising meaningful questions to find solutions for today’s burning questions in child health. To list only a few suggestions, future research could explore the perceptions of risky play among physiotherapists and allied health professions. The effects of risky play on children’s motor skills development and fitness levels could be compared to more traditional pediatric physiotherapy interventions by using randomized controlled trials. At times, pediatric physiotherapy interventions need to be provided from infancy through adolescence and even into adulthood. Not all interventions chosen are appreciated by children and their families, but sometimes perceived being boring or dull (21). In this light, it might be valuable to study children’s and their families’ adherence to and satisfaction with physiotherapy, when including risky play interventions.

Physiotherapy and risky play are environmentally friendly (90), safe, low-cost and effective ways (91, 92) to improve children’s health and well-being. Pooling both fields’ strengths can help to increase children’s awareness of the wonders of nature and life and to prepare them to accept future responsibilities towards communities, their personal health as well as planetary health (93). Nourishing a sense of belonging and connectedness (93), and reflecting on positive, playful ways of how we can build relationships and shape environments such as educational and health institutions, neighborhoods and playgrounds would be a good starting point for a healthy future worth living for humans and all other species (87).

## Data Availability

The original contributions presented in the study are included in the article/supplementary material, further inquiries can be directed to the corresponding author.
